# P30. The use of HER2 receptors status as a prognostic index for estrogen receptor positive breast cancer patients

**DOI:** 10.1186/2051-1426-2-S2-P21

**Published:** 2014-03-12

**Authors:** A Tawfeek

**Affiliations:** 1 University of Babylon College Of Medicine, Surgery, Hilla, Iraq

## Background

Tamoxifen has been a standard adjuvant hormonal treatment for estrogen receptor ER positive breast cancer, both in pre and postmenopausal women. It has been noticed that some breast cancer patients don’t respond to tamoxifen as others. In the presented study, HER2 receptor status has been studied as a probable prognostic index regarding the local recurrence & disease related mortality & to differentiate responders from non-responders to tamoxifen treatment.

## Methods

205 patients in the oncology center of Merjan Hospital, Hilla, Iraq who had mastectomy performed and histopathology done studying the ER and HER2 receptor status, all of which ER positive and of early stages (I and II) breast cancer, had been studied prospectively between the fifth of June 2010 and the 19th of June 2011 regarding the local recurrence and mortality during this period. All the patients had been given Tamoxifen tablet 20mg/day.

## Results

56 (27.3%) were HER2 receptors +ve, and 149 (72.7%) were HER2 receptor -ve . 19 (33.9%) and 9(16%) of the HER2 +ve patients had local recurrence and died due to the disease respectively during the follow up period. 30 (20.1%) and 14 (9.4%) of the HER -ve patients had local recurrence and died of the disease respectively during the follow up period.

## Conclusion

I found a significant difference in the disease local recurrence and mortality of ER+ve HER2 receptor +ve as compared with the ER+ve HER2 receptor -ve in favor of the latter and the ER+veHER2 receptor -ve had a much better prognosis.

